# Longshot or Favorite: The Ending Effect in Investment Decisions

**DOI:** 10.3389/fpsyg.2021.708211

**Published:** 2021-11-02

**Authors:** Qi Wang, JiaYuan Zhang, Cai Xing

**Affiliations:** Department of Psychology, Renmin University of China, Beijing, China

**Keywords:** ending effect, motivation, risky decision making, socioemotional selectivity theory, longshot

## Abstract

The ending effect describes the phenomenon that at the end of a series of repeated risky decision-making tasks, participants become more likely to engage in risk-taking behavior. Past research has suggested that the ending effect might be caused by a motivational shift induced by changes in time perception. Previous studies mainly tested this phenomenon in a binary decision-making setting (e.g., a decision-making task usually includes two alternatives). However, none of these prior studies included safe options and risky options that differed in risk levels. To address this knowledge gap, the present study replicated the ending effect in a repeated decision-making task that included both a safe option and risky options that differed in risk levels (*N* = 104). We found that at the end of the decision-making task, participants became more likely to engage in risk-taking and to favor the option with the highest risk. Further, we found that the investment likelihood and investment amount of high-risk options both increased significantly at the ending. In addition, a shift in favoring the safe option emerged in the noninformed condition at the end. We also found that the emotional motivation in the last round could predict the increased preference for high-risk at the ending. This study extended previous findings on the ending effect by adopting a more complex decision-making scenario and, more broadly, helped further our understanding of the psychological consequences of perceived endings.

## Introduction

Factors that influence the risky decision-making of individuals have been intensively investigated over the past decades (for a review, refer to Birnbaum, 2008). For instance, the prospect theory suggests that individuals engage in risk-taking behavior when in a loss frame of mind but show risk-aversion when in a gain frame of mind (Kahneman and Tversky, 1979). In recent years, a growing body of research has suggested that motivational factors play a role in risky decision-making behavior (Kluger et al., 2004; Florack et al., 2013). Of note, a phenomenon called the ending effect has received growing attention (McKenzie et al., 2016; Xing et al., 2018, 2019).

The ending effect, also known as the last race effect, suggests that at the end of a series of decision-making tasks, individuals tend to engage in risk-taking behavior (McKenzie et al., 2016; Xing et al., 2018, 2019). For instance, early evidence from horse racing (e.g., McGlothlin, 1956; Ali, 1977; Metzger, 1985) has shown that at the end of a racing day, bettors are more likely to bet on longshots (i.e., horses that are not likely to win, but pay a lot if they do—a high-risk option). Increased risk-taking at the end has been detected in both real-life horse racing (Ali, 1977; Asch et al., 1982; Metzger, 1985; Kopelman and Minkin, 1991) and in laboratory experiments (McKenzie et al., 2016; Xing et al., 2018, 2019). Other studies have also suggested that perceived endings may change the behavior of individuals across different domains. For example, game theory predicts an “endgame effect”, wherein individuals act in their self-interest in the last round of a repeated distribution game because there is no benefit to be obtained by cooperating (Normann and Wallace, 2012).

An earlier study has suggested that the ending effect might be reference-dependent; whether individuals show increased risk-taking behavior at the end of a decision-making task depends on whether they were relatively higher or lower than their starting (reference) point. For example, some people might have lost a lot of money at the end of a betting day. The ending effect might be driven by these individuals since prospect theory predicts that people in a loss frame of mind are more likely to engage in risk-taking behavior. However, through three well-controlled laboratory experiments, McKenzie et al. (2016) showed that regardless of previous wins or losses before the last round, participants have an increased preference for the riskier options in the last round. Such results support the idea that the ending effect is reference-independent.

More recently, based on socioemotional selectivity theory (SST, Carstensen et al., 1999; Carstensen, 2006), Xing proposed a motivational account to explain the ending effect, which posits that increased emotional motivation at the end of a decision-making task gives rise to this effect (Xing et al., 2018). SST maintains that the perception of remaining time exerts a large impact on motivation. Specifically, the perception of time of individuals becomes limited when an ending approach leading to a shift of time perception that causes them to prioritize emotionally rewarding goals (Mather and Carstensen, 2005; Carstensen, 2006).

One limitation of the previous study examining the ending effect is that only binary experimental designs have been employed. For example, in the decision-making scenario in McKenzie et al. (2016). Participants had to choose between a high-risk option and a low-risk option. Consistent with the ending effect, they found that the high-risk option was favored in the last round. In contrast, in the study of Xing et al. (2018), participants had to choose between a risky investment option (e.g., a chance of 1/6 to win) and a safe option (choose to not invest). This raises a question: in a decision scenario wherein decision-makers are provided with a safe option (choose not to bet) and risky options that vary in risk level (e.g., high-risk and low-risk options), which option will decision-makers favor? Thus far, this question has only been addressed in the early research on horse racing and the results are mixed. For instance, consistent with McKenzie et al. (2016), research focusing on on-course horse racing has demonstrated that bettors show an increased preference for betting on longshots at the end of a racing day (McGlothlin, 1956; Ali, 1977; Asch et al., 1982). In contrast, however, Johnson and Bruce (1993) found that in off-course horse racing, bettors significantly increased their overall betting amount in late races but wagered more money on favorites (i.e., horses that are expected to win and pay small if they do—a low-risk option) in late races than in earlier races. It is worth noting that in on-course betting, it is assumed that bettors will bet on every single round, and in each round, options (horses) only vary in their fixed odds. In off-course betting, however, bettors can also choose not to bet and not to wager their money. Later research using a larger dataset of both on-course and off-course horse racing found that betting on longshots increased in the last race, but the result failed to reach significance (Snowberg and Wolfers, 2010).

To the best of our knowledge, no prior studies have investigated the ending effect in a decision-making scenario that involves both a safe option and risky options that vary in risk levels. Understanding the ending effect under such conditions is critical. For example, an investor will often face a safe option (choose not to invest) and stocks of different companies which vary in risk levels (depending on potential gains and loss) at the end of a trading day. Given the significance of this research question, we conducted a laboratory experiment in an attempt to explore the boundary conditions of the ending effect. Participants were randomly assigned to the *informed* condition or the *non-informed* condition. Both conditions included 20 rounds of repeated risk-taking decisions. In the informed condition, participants were told at the beginning of the experiment that the investment task included 20 rounds, whereas, in the non-informed condition, participants did not know how many decisions they were going to make until they had finished all 20 rounds. Thus, participants were either aware (informed condition) or unaware (non-informed condition) that they were working on the last round when they were working on the 20th round. This design has been used in previous studies examining the ending effect (Xing et al., 2018, 2019), as it allows one to examine the effect of the awareness of an impending ending. Although previous findings are inconsistent, most extant evidence suggests that individuals tend to favor the riskier option in the end (e.g., McKenzie et al., 2016; Xing et al., 2018, 2019). Hence, we hypothesized that when options include a safe option and various risky options that differ in risk level, participants should invest more in the option with higher risk at the end (Hypothesis 1). Also, based on the previous findings, we hypothesized that the emotional motivation in the last round may predict increased risk-taking in the last round (Hypothesis 2).

## Method

### Participants

Prior to data collection, we performed a power analysis using G^*^Power (Faul et al., 2007) with an effect size ($\eta_{p}^{2}$ = 0.067) based on the previous research (Xing et al., 2018). The analysis results indicated that the present study required at least 72 participants to obtain a power of 0.9. A total of 105 participants were recruited from a university in Beijing, China. One participant was excluded for not finishing all measures. A total of 104 participants remained in subsequent data analyses (52 men and 52 women, *M*_*age*_ = 20.15, SD_age_ = 1.59). Participants were randomly assigned to either the informed condition (*N* = 53) or the non-informed condition (*N* = 51). They were told in advance that they would be paid based on their performance in the investment task.

### Procedures

At the beginning of the experiment, participants were given 10 tokens and were informed that (a) the payment that they received after the experiment would be equal to the number of tokens remained, (b) if they lost, they could pay either the amount that they lost in cash or help translate an English article to eliminate the loss [a manipulation to make sure that participants believed that they would lose money; (Scholer et al., 2010)]. Participants received at least ¥ 5 (RMB) even if the tokens that remained were lower than five.

Then, all participants completed 20 rounds of the investment task. In each round, participants decided whether they would invest or not. If they chose to invest, they could choose from two investment options. Option A was high-risk (rolling a die). If the die landed on the number “1” (1/6 chance), participants would win six times the token(s) they invested. However, if the die did not land on the number “1”, they would lose all the tokens that they invested (5/6 chance). Option B was low-risk (tossing a coin). If the coin landed on the number side (1/2 chance), participants would win two times the token(s) they invested. Otherwise, however, they would lose all the tokens that they invested (1/2 chance). In each round, if participants chose to invest, they could invest one to five tokens either on the low-risk or on the high-risk option. If they decided not to invest (the safe option), the experimenter still tossed the coin and the die but moved on to the next round after showing the outcome. After the decision-making session, participants were asked to recall and report their motivations on 8-point Likert-type scales (Xing et al., 2018, 2019). Specifically, participants indicated to what extent they agreed with the statement “the motivation for my decision was to satisfy my emotional need” during both the entire decision-making session and when they were making their decision in the final round. Finally, participants completed a demographic sheet prior to being paid, debriefed, thanked, and dismissed.

## Results

### Investment in Each Risk Level Changes Through the Decision Course

**Figure 1:**
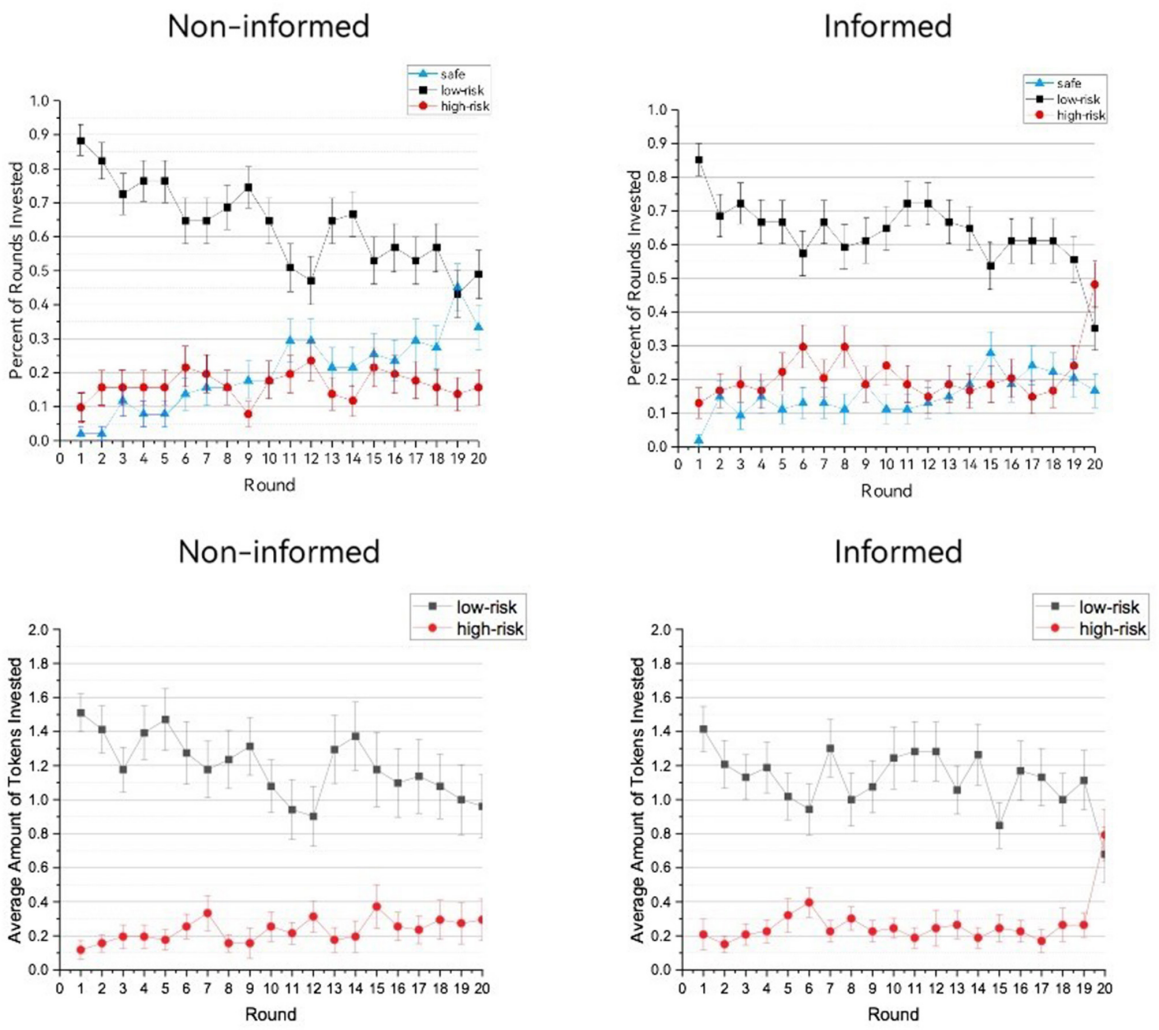
Average investment likelihood and investment amount in each of the 20 rounds of the risky decision task. Error bars represent SEs.

In the informed condition, repeated-measures ANOVAs showed that in each of the three risk conditions, the investment likelihood and investment amount significantly changed through the decision course, likelihood: *Fs*_(19,988)_ > 2.038, *ps* < 0.030, $\eta_{p}^{2}$*s* > 0.038; amount: *Fs*_(19,988)_ = 2.398, *ps* < 0.007, $\eta_{p}^{2}$*s* > 0.044. However, only in the high-risk condition, the investment likelihood (*M* ± SD = 0.472 ± 0.197) and amount (*M* ± SD = 0.793 ± 1.116) in the last round was significantly higher than the grand mean [likelihood: *M* ± SD = 0.208 ± 0.504, *F*_(1,52)_ = 17.803, *p* < 0.001, $\eta_{p}^{2}$ = 0.255, amount: *M* ± SD = 0.155 ± 0.439, *F*_(1,52)_ = 14.582, *p*s < 0.001, $\eta_{p}^{2}$ = 0.221], and higher than the previous 19 rounds, likelihood: *Fs*_(1,52)_ > 4.081, *ps* < 0.049, $\eta_{p}^{2}$*s* > 0.072 [with the only exception of the eighth round, this difference did not reach significance, *F*_(1,52)_ = 3.386, *p* = 0.071, $\eta_{p}^{2}$*s* = 0.061], amount: *Fs*(1,52) > 6.122, *ps* < 0.017, $\eta_{p}^{2}$*s* > 0.105. Identical analyses were conducted for the non-informed condition, but no increase in the last round was found, as shown in Figure 1.

### Ending Effect Score (EES)

#### The EES of Investment Likelihood

**Figure 2:**
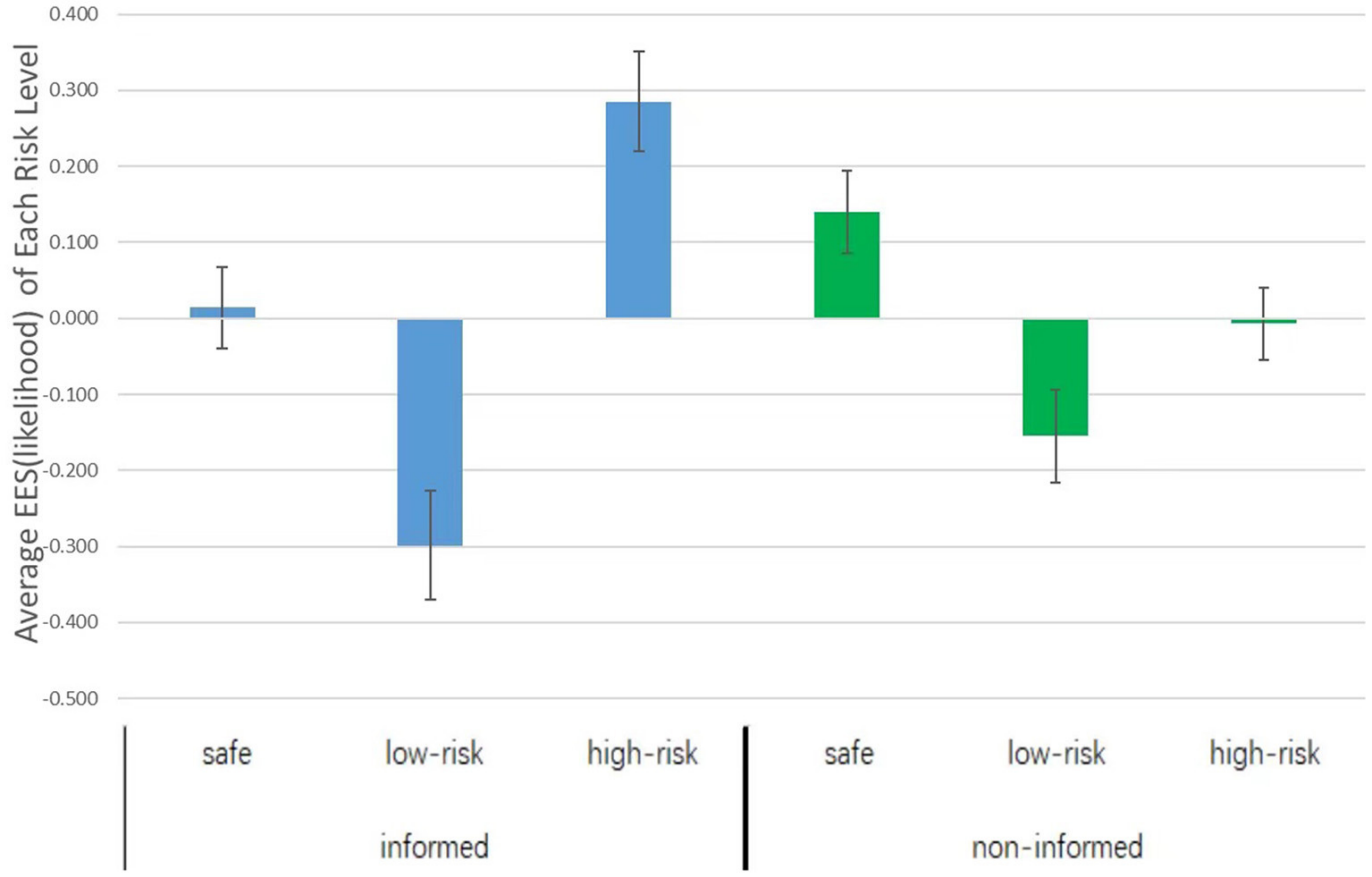
Average likelihood ending effect score (EES) of each risk level in the informed and the non-informed conditions.

We introduced the EES as it is a more straightforward and objective indicator for capturing the existence of the ending effect (Xing et al., 2018). The EES was calculated by subtracting the average investment likelihood or investment amount in all previous rounds from the investment likelihood or amount in the last round. A higher score of a certain option indicates a higher preference for the option at the end. For example, a higher EES of the high-risk option indicates more preference for the high-risk option at the end. In the informed condition, the EES of the safe option (*M* ± SD = 0.015 ± 0.396) was not significantly different from zero, *t*_(52)_ = 0.274, *p* = 0.785, *d* = 0.076, the EES of the low-risk option (*M* ± SD = −0.292 ± 0.532) was significantly lower than zero, *t*_(52)_ = −3.996, *p* < 0.001, *d* = 1.108, and the EES of the high-risk option (*M* ± SD = 0.278 ± 0.479) was higher than zero, *t*_(52)_ = 4.219, *p* < 0.001, *d* = 1.170. The results suggested that when participants knew an ending was impending, they favored the high-risk option at the ending. In contrast, in the non-informed condition, the EES of the safe option (*M* ± SD = 0.141 ± 0.392) was significantly higher than zero, *t*_(50)_ = 2.577, *p* = 0.013, *d* = 0.729, the EES of the low-risk option (*M* ± SD = −0.155 ± 0.439) was significantly lower than zero, *t*_(50)_ = −2.515, *p* = 0.015, *d* = 0.711, and the EES of the high-risk option (*M* ± SD = −0.007 ± 0.339) did not differ from zero, *t*_(50)_ = −0.152, *p* = 0.880, *d* = 0.043, see Figure 2.

#### The EES of Investment Amount

**Figure 3:**
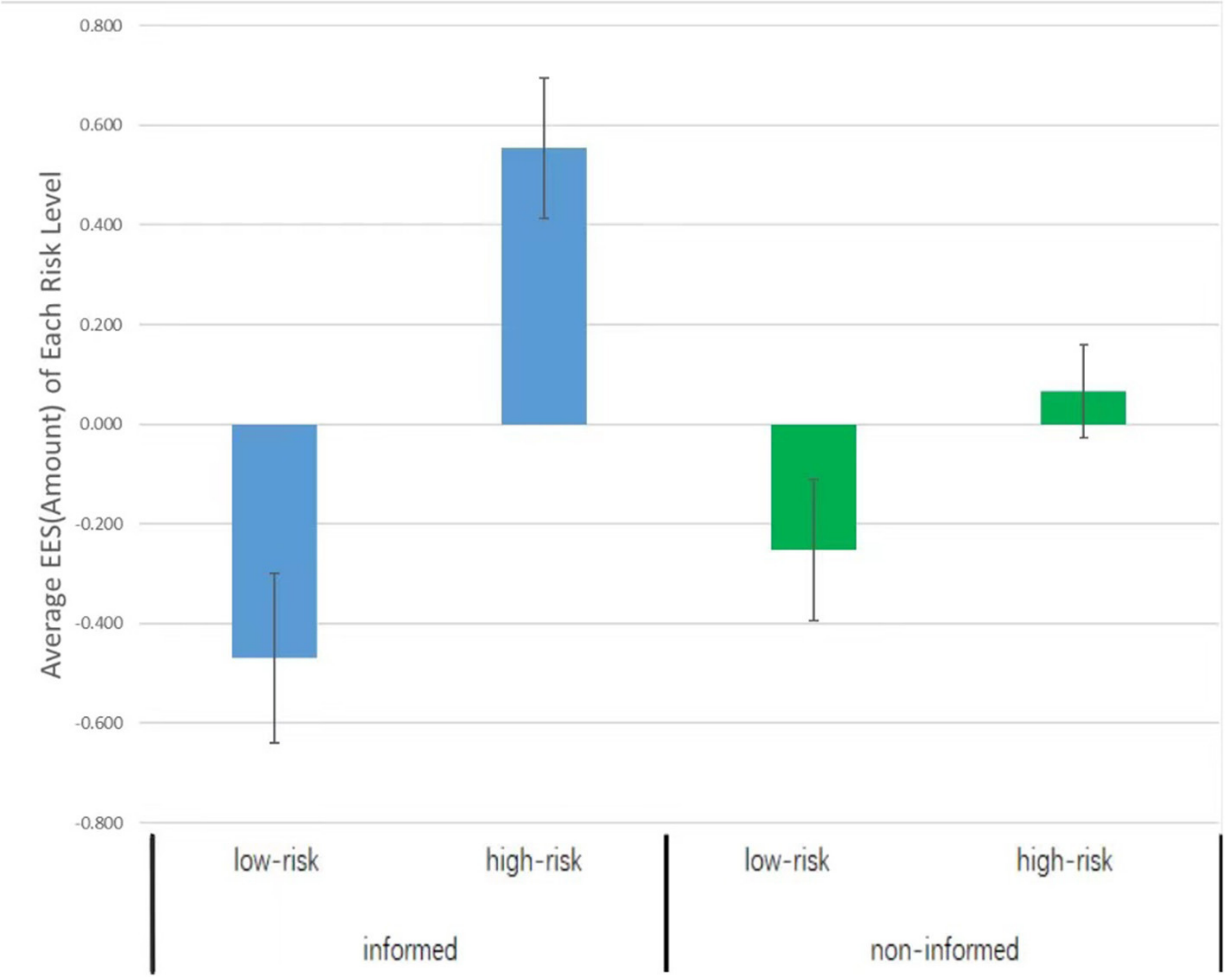
Average amount EES of each risk level in the informed and the non-informed conditions.

We introduced the EES again. Findings from one sample *t*-tests suggest that in the informed condition, the EES of the low-risk option (*M* ± SD = −0.462 ± 1.259) was lower than zero, *t*_(52)_ = −2.671, *p* = 0.010, *d* = 0.759, whereas the EES of the high-risk option (*M* ± *SD* = 0.552 ± 1.046) was higher than zero, *t*_(52)_ = 3.841, *p* < 0.001, *d* = 1.079. In the non-informed condition, both the EES of the low-risk option (*M* ± SD = −0.252 ± 1.026) and the EES of the high-risk condition (*M* ± SD = 0.066 ± 0.673) did not significantly differ from zero, low-risk: *t*_(50)_ = −1.753, *p* = 0.086, *d* = 0.496, high-risk: *t*_(50)_ = 0.701, *p* = 0.486, *d* = 0.198 (refer to Figure 3). Next, the EES of investment amount was subjected to a multivariate analysis of variance (MANOVA) with risk level as the within-subjects variable and informing condition as the between-subjects variable. The interaction between these two variables reached significance, *F*_(1,102)_ = 4.396, *p* = 0.039, $\eta_{p}^{2}$ = 0.041. Thus, we moved to simple effect analyses. Our findings indicate that the EES differed between the informed and non-informed conditions on the high-risk option, *F*_(1,104)_ = 7.873, *p* < 0.01, $\eta_{p}^{2}$ = 0.072, but not on the low-risk option, *F*_(1,104)_ = 0.866, *p* = 0.354, $\eta_{p}^{2}$ = 0.008. In the non-informed condition, the EES of low-risk options (*M* ± SD = −0.252 ± 1.026) and high-risk options (*M* ± SD = 0.066 ± 0.673) did not differ significantly, *F*_(1,102)_ = 1.800, *p* = 0.183, $\eta_{p}^{2}$ = 0.017. In the informed condition, however, the EES of high-risk option (*M* ± SD = 0.555 ± 1.037) was higher than the EES of low-risk option (*M* ± SD = −0.469 ± 1.248), *F*_(1,102)_ = 19.020, *p* < 0.001, $\eta_{p}^{2}$ = 0.186. These results suggest that participants tend to invest more of their tokens in the high-risk option when approaching an ending.

### The Role of Emotional Motivation on Increased Risk-Taking at the Ending

The second purpose of the present work was to examine the role of emotional motivation on increased risk-taking at the ending. To do so, we performed two linear regression models for the informed and non-informed conditions, respectively. In both models, last-round emotional motivation served as the predictor variable and the EES served as the dependent variable. As shown in Table 1, in the informed condition the last-round emotional motivation was positively predictive of the high-risk EES of investment amount, *B* = 0.130, *SE* = 0.061, *t*_(52)_ = 2.136, *p* = 0.037. Further, the last-round emotional motivation was negatively predictive of the likelihood of the safe option EES, *B* = −0.073, *SE* = 0.022, *t*_(52)_ = −3.340, *p* = 0.002. No other significant relationships were observed. Notably, the predictive role of motivation did not emerge in the non-informed condition. These results support our hypothesis that the shift of emotional motivation at the ending, cause participants to take higher risks rather than to make safe decisions or to satisfy themselves by taking a lower risk.

**Table 1:** Regression analyses of emotional motivation on different types of EES.

**Outcome**	**Predictor**							
	**Last-round EM (informed)**				**Last-round EM (non-informed)**			
	***B***	***t***	***CI***	***R^**2**^***	***B***	***t***	***CI***	***R^**2**^***
Safe EES_likelihood_	−0.073	−3.340**	(−0.116, −0.029)	0.180	−0.035	−1.333	(−0.087, 0.018)	0.035
Low-risk EES_likelihood_	0.017	0.531	(−0.047, 0.082)	0.006	0.000	0.006	(−0.059, 0.060)	0.000
High-risk EES_likelihood_	0.055	1.958	(−0.001, 0.111)	0.070	0.024	1.058	(−0.022, 0.069)	0.022
Low-risk EES_amount_	0.053	0.692	(0.100, 0.205)	0.009	0.078	1.144	(−0.059, 0.215)	0.026
High-risk EES_amount_	0.130	2.136*	(0.008, 0.252)	0.082	0.077	1.762	(−0.011, 0.166)	0.060

EM, emotional motivation, *p < 0.05, **p < 0.01.

## Discussion

The present study answers two fundamental questions about how the behavior of individuals changes toward the end of a series of decision-making tasks. First, in one laboratory experiment, we found that in an investment scenario that includes a safe option and risky options that vary in risk level, participants tended to take higher risk options at the ending when they knew that the end was near. Further, analyses of the emotional motivations of both the last-round and the entire round replicated previous findings that suggest the last-round emotional motivation predicts increased preference for higher risk-taking at the ending (Xing et al., 2018, 2019).

Several studies have investigated the ending effect using a binary experimental design. The present study extends previous findings by including both a safe option and risky options that vary in risk level. The computer-based risky decision tasks in McKenzie et al. (2016) and in earlier work on on-course horse racing share one thing in common: no safe option was included in the choice options. It was found that participants prefer the riskier option toward the ending. The present study showed that even when a safe option is provided, this finding remains the same: participants still prefer the riskier option on the last round. Xing et al. (2018, 2019) found that when given a safe option and a single risky option, participants showed increased preference toward the risky option on the last round. As a result, their preference toward the safe option decreased on the last round. The present study showed that when there is more than one risky option, participants prefer the riskier option on the last round. However, this shift toward increased risk-taking at the ending, which is consistent with the previous study, is accompanied by decreased preference toward the less risky options. The preference of the participants for the safe option remains stable on the last round when they perceive an impending ending.

Taken together, the increased preference for the riskiest option on the last round is robust across all studies examining on-course betting, regardless of the number of risky options provided and whether a safe option is available. This shift is accompanied by a decreased preference toward the safe option when only a safe option and a single risky option are provided (Xing et al., 2018, 2019); in contrast, when no safe option is available or more than one risky option is available, this shift is accompanied by a decreased preference toward the low-risk option (McKenzie et al., 2016 and the present study). In studies investigating off-course betting (e.g., Johnson and Bruce, 1993), the increased overall betting amount was on favorites, but not on longshots. This is inconsistent with the present study. It might be possible that individuals divide different options into two aggregated categories (i.e., the higher-risk option and the lower-risk option). The way individuals make this categorization depends on the specific decision-making situation (i.e., on-course vs. off-course betting).

Together with the motivational explanation examined in the present work, these findings may help us to better understand the driving factors behind the ending effect. Based on the SST (Carstensen, 2006), Xing et al. (2018) proposed that increased motivation toward emotional satisfaction at the ending leads to the ending effect. Following the paradigm used in Xing et al. (2018), the present study measured the emotional motivation of the participants during the last round and the entire rounds after they finished the decision-making session. Although our approach to measuring motivation may have certain limitations (discussed below), this study presents preliminary evidence for the potential role of emotional motivation before the last round on increased risk-taking at the ending. Consistent with previous work, emotional motivation in the last round could significantly predict increased preference for higher-risk options at the ending (Xing et al., 2018, 2019). Further, we found that the last-round emotional motivation also negatively predicts the preference for the safe option at the ending. As suggested by a previous study, an ending should induce a motivational shift that leads individuals to pursue emotional satisfaction (Carstensen, 2006). Across all studies, the choice for a safe option never increased on the last round when participants were aware of an impending ending. Thus, participants appear to favor a riskier, but potentially more rewarding, option to meet their need for an emotionally rewarding ending. The inconsistent finding between on-course and off-course betting suggests that the amount of risk and the size of reward needed to meet an individual's emotional satisfaction may depend on specific circumstances. This finding calls for a next step in unpacking decision situations to examine distinctive risk-taking tendencies toward an ending under different circumstances.

In the non-informed condition, the preference of the participants for the safe option generally increased and their preference for the low-risk option tended to decrease, while their preference for the high-risk option remained stable. The risk preference of the participants shifted between low-risk and safe options was more pronounced around the 10th round and on the 19th round than on other rounds. Thus, it appears that there was a shift in favoring the safe option in the non-informed condition at the end. One possibility is that participants treated every 10 rounds as a stage. For instance, participants may have viewed the 10th round and 20th round as the end of each stage, leading to shifts in risk-taking behavior. To address this concern, future studies examining the ending effect should avoid using multiples of 10 or 5 as the number of rounds or use a different number of rounds as the control group (Effron et al., 2015). Another possible explanation is that as the decision task continues, after several trials, people tend to become more conservative because generally, people are risk aversive (Larrick, 1993; Benartzi and Thaler, 1995). This possibility also demands further examination to investigate the time course of change in risky decision-making when no end is perceived initially.

While the results presented here further our understanding of the ending effect, this study had a few limitations. First, the emotional motivation was measured by self-report questions after participants finished all decision tasks. We chose this approach to measure motivation because it allowed participants to complete the 20 rounds of investment decisions without being affected by measurements of motivation. Therefore, this study could accurately reveal changes in investment decisions of participants across the 20 rounds. However, reporting emotional motivation in this manner may be biased. For instance, participants may have inaccurately recalled their motivations and/or used their replies to justify their investment decisions. Such biases may invoke serious concerns when interpreting the motivational result. A fruitful extension would involve using other types of measurement to assess emotional motivation, measuring the emotional motivation prior to the decision task, and comparing it with the measurement after the decision task. Second, the participants included were college students, who, in general, are less experienced in terms of gambling (Ciccarelli et al., 2017). Consequently, their behavioral pattern, mindset, and motivations may have differed from that of experienced gamblers (Nigro et al., 2017). An interesting direction would be to study experienced gamblers and compare their risk-taking behaviors to novice counterparts.

In sum, the present study complements existing work examining the ending effect in risky decision-making. A previous study examining the ending effect in risky decision-making has shown that as individuals approach an ending, they prefer a high-risk option over a low-risk option (McKenzie et al., 2016) and a risky option over a safe option (Xing et al., 2018, 2019). The present study complements existing work by showing that as individuals approach an ending, they would prefer to invest in the high-risk option over the low-risk option and the option of not to invest. Consistent with previous studies, the results in the present study support the idea that this shift in risk preference could be explained by the need for an emotionally rewarding ending.

## Data Availability Statement

The raw data supporting the conclusions of this article will be made available by the authors, without undue reservation.

## Ethics Statement

The studies involving human participants were reviewed and approved by IRB of the Psychology Department at Renmin University of China. The patients/participants provided their written informed consent to participate in this study.

## Author Contributions

QW is in charge of designing the research protocol, writing the original draft, helping with data collection, data analysis, manuscript reviewing, and editing. JZ is helping with data collection and responsible for results visualization. CX is responsible for designing and correcting the research protocol including conceptualization, methodology, also in charge of manuscript reviewing, editing, supervision, and the whole project administration. All authors contributed to the article and approved the submitted version.

## Funding

This research was supported by National Natural Science Foundation of China Grant 71873133 awarded to CX.

## Conflict of Interest

The authors declare that the research was conducted in the absence of any commercial or financial relationships that could be construed as a potential conflict of interest.

## Publisher's Note

All claims expressed in this article are solely those of the authors and do not necessarily represent those of their affiliated organizations, or those of the publisher, the editors and the reviewers. Any product that may be evaluated in this article, or claim that may be made by its manufacturer, is not guaranteed or endorsed by the publisher.
